# Endoplasmic Reticulum Stress and Reactive Oxygen Species in Plants

**DOI:** 10.3390/antiox11071240

**Published:** 2022-06-24

**Authors:** Jiajian Cao, Chunhua Wang, Ning Hao, Toru Fujiwara, Tao Wu

**Affiliations:** 1 College of Horticulture, Hunan Agricultural University, Changsha 410128, China; caojiajian@hunau.edu.cn (J.C.); chwang@hunau.edu.cn (C.W.); 2 Key Laboratory for Evaluation and Utilization of Gene Resources of Horticultural Crops (Vegetables, Tea, etc.), Ministry of Agriculture and Rural Affairs of China, Changsha 410128, China; 3 Engineering Research Center for Horticultural Crop Germplasm Creation and New Variety Breeding, Ministry of Education, Changsha 410128, China; 4 Graduate School of Agricultural and Life Sciences, The University of Tokyo, Tokyo 113-8657, Japan; haoning0925@hotmail.com (N.H.); atorufu@g.ecc.u-tokyo.ac.jp (T.F.)

**Keywords:** endoplasmic reticulum stress, reactive oxygen species, redox stress, redox homeostasis, plants

## Abstract

The endoplasmic reticulum (ER) is a key compartment responsible for protein processing and folding, and it also participates in many signal transduction and metabolic processes. Reactive oxygen species (ROS) are important signaling messengers involved in the redox equilibrium and stress response. A number of abiotic and biotic stresses can trigger the accumulation of unfolded or misfolded proteins and lead to ER stress. In recent years, a number of studies have reported that redox metabolism and ROS are closely related to ER stress. ER stress can benefit ROS generation and even cause oxidative burden in plants, finally leading to oxidative stress depending on the degree of ER stress. Moreover, ER stress activates nicotinamide adenine dinucleotide phosphate (NADPH) oxidase-mediated ROS signaling, increases antioxidant defense mechanisms, and alters the glutathione (GSH) redox state. Meanwhile, the accumulation of ROS plays a special role in inducing the ER stress response. Given these factors, plants have evolved a series of complex regulatory mechanisms to interact with ROS in response to ER stress. In this review, we summarize the perceptions and responses of plant ER stress and oxidative protein folding in the ER. In addition, we analyze the production and signaling of ROS under ER stress in detail in order to provide a theoretical basis for reducing ER stress to improve the crop survival rate in agricultural applications.

## 1. Introduction

The endoplasmic reticulum (ER) is a subcellular compartment composed of a network of tubular membranes, responsible for protein processing and folding and also involved in many signal transduction and metabolic processes, including lipid biosynthesis and ion storage [1]. In eukaryote cells, the ER provides a place for a bulk of nascent synthesized proteins, where they could be post-translationally modified, folded, and/or assembled into protein complexes [2]. However, the process of protein biosynthesis and modification in the ER is error-prone and could be adversely affected by stressful environments, such as high temperatures, salinity, and bacterial pathogen infection, resulting in unfolded or misfolded protein loads [3]. Under these abiotic and biotic stress conditions, plants accumulate a large number of unfolded or misfolded proteins which do not have a particular function in the ER lumen, leading to ER stress [4]. To eliminate the accumulation of misfolded proteins in the ER, the ER processes quality control (QC) systems to monitor protein folding and ensure its fidelity. One of the most conserved parts of ERQC systems is the unfolded protein response (UPR), which promotes the folding process by activating ER chaperone genes [5]. In addition, ER-associated degradation (ERAD) and autophagy are considered to play an important role in ER homeostasis reconstruction and contribute to the degradation of misfolded proteins [6]. When the stress exceeds a threshold and the ER’s compensatory mechanisms are no longer able to maintain their function, programmed cell death (PCD) occurs (which is specifically known as vacuole cell death) [7].

In recent years, the regulatory network and signaling mechanism of the plant ER stress response have been extensively studied. It has been reported that redox metabolism and reactive oxygen species (ROS) are closely related to ER stress. ER stress is found to directly generate ROS signaling, modify the redox status, and modulate Arabidopsis antioxidant defense [8]. The role of ROS is crucial for abiotic/biotic stresses, and they are considered to have dual roles in plant biology, acting as signaling molecules at basal non-toxic levels, while causing damage to DNA, RNA, proteins, and membranes at excessive toxic levels as by-products of aerobic metabolism [9]. Therefore, many cellular antioxidative systems prevent the toxic effects of ROS, mainly through a series of antioxidant enzymes and antioxidants for removal or detoxification [9]. The key enzymatic antioxidant superoxide dismutase (SOD) is present in all forms of life to balance the ROS levels of different organisms [9]. In addition to SOD, catalase (CAT), peroxidase (PER), glutathione peroxidase (GPX), and other antioxidant enzymes in plants have been widely studied [9,10]. Major non-enzymatic antioxidants have also been found in plant cells, mainly metabolites and vitamins, including glutathione (GSH), carotenoids, flavonoids, ascorbic acid (AsA), and tocopherol [9,10]. The ratio of GSH to the oxidized form glutathione disulfide (GSSG) in the ER is reported to be important for correct protein folding since it affects the stability of disulfides [11]. Moreover, the ER can accumulate Ca^2+^; luminal Ca^2+^ can promote protein folding/processing functions [12], and Ca^2+^ is reported to activate ROS production and, in turn, be regulated by ROS signals [13,14,15].

In recent years, progress has provided deep insights into the interrelationship of ER stress and ROS with redox signaling, as well as their important roles in multiple human diseases [16,17,18,19]. Recently, studies on ER stress and stress-induced ROS signals in plants have increased [1,2,6,9,20,21,22]. These publications review ER stress perception, signaling, and responses, as well as stress-induced ROS generation, compartmentalization, and homeostasis in plants. However, there are very limited studies on the interaction between ROS and cellular redox homeostasis associated with ER stress in plants. In this review, we summarize the regulatory networks and signaling mechanisms of the ER stress responses and related ROS in plants.

## 2. Perceptions and Responses under Plant ER Stress

The ER is the primary organelle in charge of protein folding, biosynthesis, trafficking, and post-translational modifications. With the assistance of the cooperative actions of ER chaperone genes and folding enzymes, polypeptides of secretory and transmembrane proteins enter the ER lumen through the Sec61 translocon to perform folding and post-translational modification including *N*-linked glycosylation and disulfide bond formation [23]. Binding proteins (BiPs) are heat shock 70 proteins, one of a large number of ER chaperones that bind to nascent proteins entering the ER and prevent them from aggregating [7]. Protein disulfide isomerase (PDI), the first identified protein folding enzyme catalyst, is a dithiol-disulfide oxidoreductase capable of reducing, oxidizing, and isomerizing disulfide bonds [24]. Calnexin (CNX)/calreticulin (CRT) are lectin-like chaperones that identify *N*-linked oligosaccharides and maintain unfolded glycoproteins in the ER [2]. ER oxidoreductase 1 (ERO1) cooperates with PDI family proteins mediating oxidative folding with disulfide bond formation [24]. After these folding and posttranslational modification mechanisms, the obtained mature proteins are then transported outside the ER.

**Figure 1:**
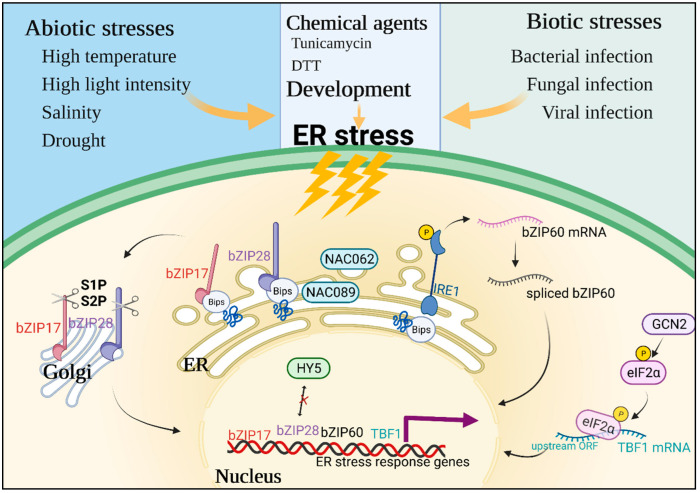
An overview of perceptions and responses under plant ER stress. Abiotic/biotic stresses and some chemical agents, as well as developmental processes, are able to trigger ER stress. Under ER stress, many transcription factors are up-regulated, which can be translocated into the nucleus and then target downstream ER stress response genes. Meanwhile, some protein modifications are activated, such as the phosphorylation of IRE1 and elF2α which are reported to respond to ER stress. ER, endoplasmic reticulum; bZIP17/28/60, bZIP17 transcription factor 17/28/60; NAC062/089, NAC domain-containing protein 062/089; TBF1, TL1-binding factor; BiPs, binding proteins; IRE1, inositol-requiring enzyme 1; GCN2, general control non-repressible 2; elF2α, translation initiation factor 2α; ORF, open reading frame.

When plants are faced with unsuitable conditions including abiotic and biotic stresses, the ER folding machinery is impaired and the accumulation of unfolded proteins in the ER causes ER stress and activates the UPR (Figure 1) [7]. Specifically, abiotic (e.g., high temperature, high light intensity, salinity, and drought) and biotic stresses (e.g., bacterial, fungal, or viral plant pathogen infection), as well as some developmental processes, can lead to the accumulation of unfolded proteins and induce ER stress, resulting in activation of the UPR [25]. Additionally, some laboratory chemicals can also trigger ER stress. For example, tunicamycin acts as an inhibitor of *N*-glycan chain synthesis, and DTT acts as a reductant that disrupts disulfide bond formation, both of which cause unfolded polypeptide chains to accumulate in the ER [4]. These changes are firstly sensed by the special sensor proteins anchored on the ER membrane, which mainly include two categories in plants: ER membrane-associated transcription factors and the RNA splicing factor [26]. The bZIP family transcription factors bZIP17 and bZIP28 play an important role in sensing ER stress through interaction with BiPs. Under ER stress, BiPs interact with unfolded or misfolded proteins and dissociate with bZIP17 and bZIP28, resulting in two bZIP proteins transported from the ER to the Golgi. In the Golgi, two bZIP proteins are hydrolyzed and cleaved by Golgi-associated site-1 and site-2 proteases (S1P and S2P), releasing their N-terminal cytosolic portions which relocate into the nucleus. Then, nuclear bZIP17/28 can bind to target promoters of stress-response genes and activate their expression, restoring the ER equilibrium [2,26,27]. For example, Arabidopsis *bZIP17* was highly up-regulated under salinity-related ER stress and translocated to the nucleus to promote the expression of downstream stress-related genes [28]. Additionally, plants with truncated Arabidopsis *bZIP17* (*bZIP17*∆C) and the stress-inducible RD29A promoter showed enhanced resistance to salt stress compared to the wild-type [28], while the *bzip17* T-DNA mutant was highly sensitive to salt stress [29]. Moreover, it has been reported that the bZIP protein long hypocotyl 5 (HY5), a positive regulator in light signaling transduction, competitively inhibits the interaction of bZIP28 and suppresses the UPR pathway, causing ER stress sensitivity upon high light intensity [30]. In addition to bZIP protein families, several ER- or plasma membrane-tethered NAC transcription factors were found to mediate ER stress and modulate the UPR [26]. Both Arabidopsis *NAC089* and *NAC062* knockdown plants were sensitive to ER stress, and the expression of UPR downstream genes induced by ER stress was impaired in plants [31,32]. Another type of ER stress sensor is inositol-requiring enzyme 1 (IRE1), which acts as a serine/threonine kinase and has endoribonuclease (RNase) activity [26]. IRE1 is a highly conserved protein. Monocot plants such as rice have a single *IRE1* gene. Dicots usually have two homologs, *IRE1a* and *IRE1b*, and Brassicas additionally have *IRE1c* [25,33]. Under ER stress, luminal domains of AtIRE1s sense this change, and then cytoplasmic domains of AtIRE1s splice *AtbZIP60* mRNA [33]. The splicing of AtbZIP60 can enter the nucleus to promote the expression of ER stress response genes [2]. Additionally, IRE1 also mediates the selective degradation of certain mRNAs encoding secretory pathway proteins and negative regulators of the ER stress-induced autophagy, a mechanism known as regulated IRE1-dependent decay of mRNAs (RIDD) [27,34]. Both Arabidopsis and *Chlamydomonas reinhardtii ire1s* mutants showed significant sensitivity to ER stress, demonstrating that *IRE1s* and RIDD play a role in ER stress alleviation [35,36].

Recently, the phosphorylation of eukaryotic translation initiation factor 2α (eIF2α), which is dependent on general control non-repressible 2 (GCN2), has been demonstrated to be another key component in response to ER stress, further underlining the similarities in the processes of UPR signaling among animals and plants [25,33]. Moreover, mammals possess another protein kinase, the protein kinase RNA-like ER kinase (PERK), to accompany these processes, but it is absent in plants [33]. Arabidopsis and tobacco encode one and two GCN2 kinases, respectively, which are stimulated by a range of ER stress-related abiotic and biotic elicitors, such as drought, cold, nutrition deficiency, sulfonylurea herbicide, and bacterial infection [37,38,39]. Additionally, AtGCN2-mediated phosphorylation of eIF2α was shown to promote translational de-repression of mRNAs through re-initiation of upstream open reading frames (ORFs), such as TL1-binding factor 1 (TBF1), which regulates a subset of secretory pathway-related components to regulate pathogen immunity [39,40].

## 3. Oxidative Protein Folding in the Plant ER

**Figure 2:**
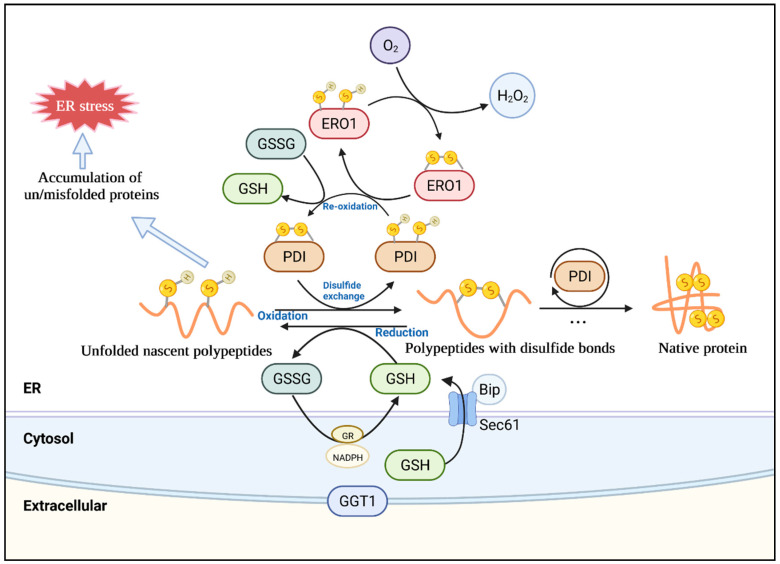
A simplified schematic of oxidative protein folding. The oxidative protein folding processes are presented, mainly including various oxidative modifications of the nascent protein thiol (-S-H) and the production of H_2_O_2_ in these reactions. Since the protein folding process in the ER is error-prone, it may be disturbed by adverse conditions, resulting in the accumulation of unfolded or misfolded proteins, leading to ER stress. ER, endoplasmic reticulum; PDI, protein disulfide isomerase; GSH, glutathione; GSSG, glutathione disulfide; ERO1, ER oxidoreductase 1; GR, glutathione reductase; NADPH, nicotinamide adenine dinucleotide phosphate; GGT1, γ-glutamyl transpeptidase 1; Bip, binding protein; Sec61, protein transport protein Sec61.

In addition to the above-mentioned correct protein folding events and incorrect protein response events in the ER, the ER is also the post-translational modification site, that is, the site of oxidative folding with disulfide bond formation (Figure 2). Therefore, the direct connection of oxidative protein folding in the ER should first be considered when investigating the interplay between ER stress and ROS. The protein folding to form a disulfide bond (R-S–S-R) in the ER lumen is named oxidative protein folding, which requires the oxidation of thiol (-S-H) groups [24]. The disulfide-thiol reactions require some important catalysts, such as the PDI family. The thioredoxin-like PDI enzymes oxidize the targeted proteins to generate disulfide bridges. PDIs are members of the thioredoxin superfamily and consist of two thioredoxin domains (a and a′) with catalytic CXXC motifs and two inactive domains (b and b′) with thioredoxin-like folding structures [24]. There is a wide variety of PDIs in higher eukaryotes to specifically target potential substrate proteins. For instance, a large-scale genomic study and BLAST analysis across photosynthetic species revealed that PDIs range from 4 in the red algae *Cyanidioschyzon merolae* to 22 in *Glycine max* [11,41]. Among higher plants, 22 PDI orthologs were found in Arabidopsis, 19 ortholog sequences were found in rice, and 22 ortholog sequences were found in maize [41]. In Arabidopsis, these PDIs were phylogenetically resolved into 10 family groups, most of which have ER retention signals [41,42]. Additionally, the recombinant proteins expressed in *E. coli* demonstrated that soybean GmPDIL1 and GmPDIL2, as well as wheat TaPDIL1 and TaPDIL2, have disulfide isomerase activity and oxidative refolding activity against RNase A [43,44].

Besides PDIs, additional factors are also necessary for the catalysis of disulfide bonds. Disulfide bridges are generated between PDI proteins and thiol groups of target proteins, during which two thioredoxin domains of the PDI family are reduced, thus requiring an oxidative equivalent to enable oxidative folding [24]. However, most PDI family proteins cannot re-oxidize their active centers, and hence other oxidizing components or systems are required to catalyze a new round of oxidation processes [4]. Upstream of PDIs, ERO1 contributes to the re-oxidation of PDIs and is closely connected with the surface of the ER membrane [45]. In this process, ERO1 helps produce hydrogen peroxide (H_2_O_2_) which is the result of electron transport from target proteins to O_2_ via thiol exchange [4]. Ero1p was first identified in yeast, and later two homologs (Ero1α and Ero1β) in humans were discovered, all of which preferentially oxidize PDIs but not other PDI family members [46]. In plants, Arabidopsis (AtERO1 and AtERO2), rice (OsERO1), and soybean ERO1 (GmERO1a and GmERO1b) were found to be homologs of the yeast Ero1p [4,24]. These proteins show conserved transmembrane domains at the N-terminal side, which anchors proteins to the ER membrane [4,24]. Furthermore, GmERO1 has a broad substrate selectivity, oxidizing five PDI family proteins, including GmPDIL1, GmPDIM, GmPDIS1, GmPDIS2, and GmPDIL7 [46]. Additionally, ERO1s are known to catalyze asymmetric oxidation in the two active centers of PDI. For example, GmERO1a primarily oxidizes the a′ active domain of GmPDIM, and the disulfide bond is transported to the active domain of GmPDIM, and then oxidizes the a and a′ active domains of GmPDIL2 [24]. Moreover, GmPDIM and GmPDIL2 oxidized by GmEro1a can enhance the oxidative folding of denatured d RNase A in vitro [46]. EROs are regulated by negative feedback. For instance, the oxidation of active domains in turn limits the activity of ERO1 and prevents further oxidation when the ER lumen becomes oxidized, while the reduction conditions activate ERO1 [4]. This type of manipulation is critical for preventing excessive ER lumen oxidation. In particular, besides thiol oxidation, H_2_O_2_ generated by ERO1 is also transferred into the ER lumen to further oxidize the surroundings [47].

In addition to enzymic pathways for PDI re-oxidation, various small-molecule oxidants have been discovered for unfolded reduced proteins. GSSG was known as an instant oxidant for protein thiols. GSH functions as an electron donor, reducing disulfide bonds generated in cytoplasmic proteins to cysteine, and it is changed into GSSG in this process. Thus, the GSH-GSSG redox pair status is important for the establishment of native disulfide bonds during protein folding and the avoidance of stress, which is an indicator of thiol-disulfide homeostasis in the ER [11]. The redox-sensitive green fluorescent protein (roGFP) sensor determined that the electrochemical reduction potential of GSH in the ER was −208 mV, much higher than that in the cytosol (−300 mV), indicating the GSH in the ER has moderate reducing capability [45,48]. Therefore, GSH-mediated reduction leads to the formation of its dimeric oxidized form GSSG. After oxidation, GSH could be re-regenerated by glutathione reductase (GR) using nicotinamide adenine dinucleotide phosphate (NADPH) as an electron donor. However, GR activity is absent in the ER, resulting in a highly oxidized environment favorable for disulfide bond production [49]. As mentioned above, tunicamycin and DTT as laboratory agents can cause ER stress. Previous studies have shown that tunicamycin-induced ER stress caused GSH accumulation, upregulated some glutathione-related enzymes, and affected the cell’s redox state in Arabidopsis [50]. Additionally, the GSH biosynthesis genes *GSH1* and *GSH2* were also induced by ER stress with different concentrations of tunicamycin, but the gene, *γ-glutamyl transpeptidase 1* (GGT1), related to apoplastic GSH degradation was induced at the same time [50]. This may be due to the fact that GGT1-mediated degradation helps recycle amino acids for GSH re-generation in the absence of GR in the extracellular matrix [51]. Additionally, Arabidopsis *ggt1* mutant exhibited an elevated abundance of a variety of antioxidant enzymes compared to the control plant via biochemical and quantitative proteomics analysis, acting as a constitutive alert response, which is similar to the response of plants to abiotic and biotic stresses [52]. This finding indicates that GGT1 participates in redox signaling from the extracellular space to internal regions, delivering redox signals required for plant adaptation to different environments. Additionally, DTT acts as a disulfide exchanger and is usually utilized to create ER stress. However, it also produces reductive stress not only in the ER but possibly in all compartments of the cell, such as the chloroplasts and mitochondria [4]. Thus, the use of DTT reducing agents to investigate ER stress and UPR reactions requires further consideration, as redox signals or metabolic pathways regulated by oxidoreductases in these different compartments may interfere with each other.

The ER provides a unique environment for GSH homeostasis. ERO1 catalyzes disulfide bond formation to folding substrates via PDIs and further excites GSH import by GSH oxidation to GSSG, and ERO1 can be regulated by the negative feedback of disulfides when the ER becomes overoxidized [53]. GSH has been reported to facilitate diffusion into the ER via the Sec61 protein transduction channel and is regulated by ERO1-associated BiP in yeast [53]. Under ER stress, GSH trafficking into the ER was promoted through the activation of both ERO1 and cellular GSH levels; when GSH increased in the lumen, the reductive activation of ERO1 was activated, triggering H_2_O_2_-dependent BiP oxidation, and feedback inhibited GSH transport [53]. Thus, the GSH and GSSG ER-to-cytosol fluxes and the activation of ERO1 maintain the ER redox homeostasis in yeast, but there is little knowledge on similar regulating systems in plants, and this subject requires further investigation.

## 4. Production and Signaling of ROS under ER Stress

**Figure 3:**
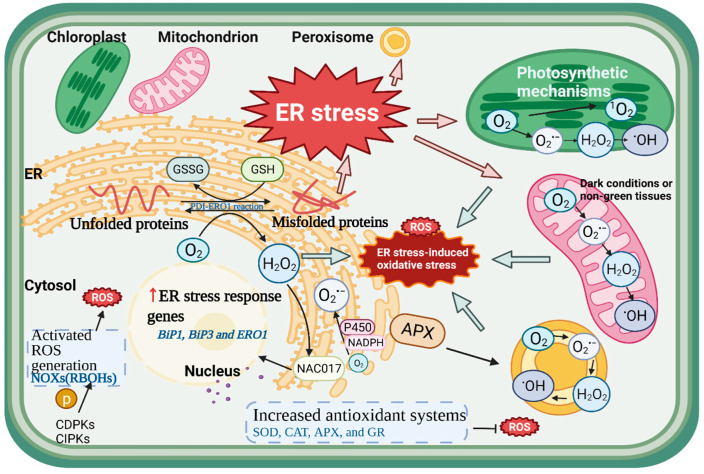
An overview of the linkage and crosstalk among ROS, oxidative stress, and ER stress in plants. ROS can be generated in different cellular compartments, including the ER, mitochondria, chloroplasts, and peroxisomes, and ROS can act as signaling molecules between the ER and other organelles. Under adverse environments, unfolded or misfolded proteins accumulate in the ER leading to ER stress while also activating oxidative stress. Meanwhile, the enzymic complexes NOXs related to ROS generation are also activated, thus causing an increase in antioxidant systems to scavenge ROS. ER, endoplasmic reticulum; ROS, reactive oxygen species; GSH, glutathione; GSSG, glutathione disulfide; PDI, protein disulfide isomerase; ERO1, ER oxidoreductase 1; BiP1, binding protein 1; NAC017, NAC domain-containing protein 017; P450, cytochrome P450 reductase; NADPH, nicotinamide adenine dinucleotide phosphate; NOXs(RBOHs), NADPH oxidases (respiratory burst oxidases); CIPKs, B-like protein-interacting protein kinases; CDPKs, Ca^2+^-dependent protein kinases; SOD, superoxide dismutase; CAT, catalase; GR, glutathione reductase.

The term ROS mainly refers to oxygen free radicals and their derivatives, including H_2_O_2_, superoxide (O_2_^•−^), singlet oxygen (^1^O_2_), and the hydroxyl radical (^•^OH), which are generated by numerous cellular metabolisms in distinct cellular compartments, including the ER, mitochondrion, chloroplast, and peroxisome especially during stress conditions [9]. In the major cellular compartments, the production of ROS from molecular O_2_ generally creates O_2_^•−^ and H_2_O_2_, which can be further dismutated to ^•^OH. Numerous studies have indicated that ROS play a dual role in plant biology as both harmful products of physiological metabolisms and important regulators of growth and development [20]. In general, ROS are naturally produced in plants as byproducts of aerobic metabolism. When exposed to adverse and stress conditions, however, the over-production of ROS breaks the balance between ROS accumulation and clearance, resulting in oxidative damage to biomolecules, which causes cellular damage and death. Most types of biotic or abiotic stress impair the cell metabolic equilibrium, resulting in increased ROS generation, e.g., pathogen infection, drought, salinity, and temperature stress, as well as a high light intensity [10]. The production of ROS is the primary part of the reaction to disturbances, and its main role is to restore homeostasis, which can be thought of as a signal of cellular stress. When ROS are over-accumulated, the cell’s antioxidant capacity is overwhelmed, leading to an oxidative reaction, namely oxidative stress. In recent years, the relationship between ER stress and oxidative stress has been widely concerned in human diseases [16,19,54], but there are few reports in plants. Thus, it is worth exploring the linkage and crosstalk among ROS, oxidative stress, and ER stress in plants (Figure 3).

### 4.1. The Generation of ROS in the ER

The ER can be regarded as a subcellular compartment for ROS generation. O_2_^•−^ is produced in the ER as a by-product of oxidation and hydroxylation processes involving the electron donor NADPH and cytochrome P450 reductase [55]. NADPH oxidases (NOXs) carry an electron from cytoplasmic NADPH to create O_2_^•−^ in the plasma membrane, which is then transformed to H_2_O_2_. Cytoplasmic NADPH regulates the status of thiols/disulfides and contributes to the synthesis of reducing substrates for antioxidant enzymes during ROS breakdown and metabolism. Thus, it is the core component in the cytoplasm to maintain redox homeostasis, and it also provides electrons to the plasma membrane NOXs in the form of ROS-producing enzymes [56]. NOXs are also known as respiratory burst oxidases (RBOHs) in plants. Arabidopsis RBOHD and RBOHF were specifically localized in the plasma membrane, but tobacco RBOHD was found in lipid rafts, which are membrane microdomains in tobacco cells that can be linked to other membrane components [57,58]. It appears that different subcellular localization of RBOHs are crucial to their activity. Cytochrome P450 reductase, as part of the microsomal monooxygenase system, is commonly reported in mammals as one of the main sources of ROS in the ER [19]. The electron transfer efficiency or coupling degree from NADPH to p450 is usually 50–60%, and sometimes as low as 0.5–3.0%, while this electron leakage plays a crucial role in ROS formation [19]. In plants, Arabidopsis *early lesion leaf 1* (ELL1) encodes a cytochrome P450 monooxygenase, which is located in the ER. Additionally, the Arabidopsis *ell* mutant showed excessive ROS accumulation in leaves inducing DNA damage and cell death [59]. Furthermore, according to the above, a large number of experiments have shown that oxidative protein folding in the ER is an important source for intracellular ROS production. We will elaborate on this point in the following. After ERO1 accepts electrons from PDI, it transfers electrons to atomic oxygen to produce H_2_O_2_, the main ROS generated in the ER cavity [4]. It was reported that about 25% of ROS in yeast came from the formation of a disulfide bond mediated by Ero1p, which is the amount of H_2_O_2_ produced in the process of ER oxidative protein folding [60]. In addition to ROS production, the ER and cytoplasmic matrix play key roles in redox signaling in plant cells. Normally, ROS signaling from different organelles regulates gene expression in the nucleus through cytoplasmic components [61]. For example, the Arabidopsis transcription factor NAC017 was reported to be localized in the ER, and the N-terminal region can migrate to the nucleus when responding to H_2_O_2_ stress treatment, leading to downstream transcriptional changes [62]. On the one hand, ROS are generated during the ER protein folding process; on the other hand, ROS in turn regulate the ER protein folding process. It was reported that some parts of the UPR during mammalian disease could be activated by some oxidants, including peroxides, oxidation by-products, and metal ions [54]. Additionally, different ROS types act on diverse UPR components and trigger various UPR reaction intensities; that is, the ROS-induced oxidative stress strength and location may determine whether it is sufficient to induce strong ER stress [54]. Therefore, when discussing the relationship between ER and ROS, it is hard not to explore the response of ER stress to ROS.

### 4.2. ROS Signaling Triggered by ER Stress

There is no doubt that ER stress triggers ROS signaling. In Arabidopsis, salt stress and tunicamycin-induced ER stress stimulated the production of H_2_O_2_, as well as the peroxidation of lipids and proteins, resulting in oxidative stress [8]. Additionally, in these processes, some genes related to ER stress were activated, including bZIP family transcription factors, *BiP1*, *BiP3,* and *ERO1*. Consistent with these results, the activity and the synthetic gene expression of *NOXs*, which is the key enzyme in ROS generation, were also up-regulated by ER stress [8]. Apoplastic ROS signaling is often connected with the enzymic complexes NOXs (RBOHs), especially under ER stress. It has been confirmed that RBOHD and RBOHF are essential for ROS production under ER stress in *Arabidopsis thaliana* [57]. After 48 h of tunicamycin treatment, an increased level of H_2_O_2_ was observed in Arabidopsis wildtype tissues but not in the *rbohd rbohf* mutant, suggesting that RBOHD and RBOHF are needed for the production and accumulation of H_2_O_2_ under ER stress [57]. However, when treated with the NOXs inhibitor diphenyleneiodonium, a decreased accumulation of H_2_O_2_ was observed in Arabidopsis [8]. Plant RBOHs have strong amino acid sequence similarity in the C-terminus, containing cytosolic FAD- and NADPH-binding domains, whereas they have more variability in N-terminal regions, mainly containing two EF-hand domains; as long as Ca^2+^ is bound to at least one of the EF motifs, O_2_^•−^ production can be initiated, indicating that Ca^2+^ signaling is closely linked with ROS biosynthesis under ER stress [4]. It has been shown that enhanced NOXs activity was found in *Arabidopsis thaliana* when treated with a Ca^2+^ ionophore, ionomycin, resulting in increased ROS generation [4]. In addition to Ca^2+^ binding, NOXs also need phosphorylation to become sufficiently activated, including the participation of Ca^2+^-dependent protein kinases (CDPKs) and calcineurin B-like protein-interacting protein kinases (CIPKs) [4]. In addition to Ca^2+^, other early messengers such as nitric oxide (NO) also participate in the ER stress response and transmit signals to regulate plant growth, development, and stress resistance by analyzing and interpreting external stresses [14]. It was reported that Arabidopsis lacking *RBOHD* or *RBOHF* could not accumulate NO to respond to pathogen attacks [63,64], indicating that there was a feedback loop between NO and ROS. Additionally, similar to ROS, reactive nitrogen species (RNS) as active molecular redox radicals might interact with ROS to form additional molecules. For example, superoxide nitrite (ONOO^−^) is generated by the interaction of NO and O_2_^•−^ [65]. Whether ROS, RNS, and their derived complexes function under ER stress is worth exploring. Besides ROS production, previous research has revealed that various antioxidant defense mechanisms regulated the imbalance of the redox status during ER stress. One study showed that salinity, tunicamycin, and their combination induced ER stress accompanied by an increase in antioxidant systems, including the major ROS scavenging enzymes SOD, CAT, APX, and GR [8]. However, this increment was uneven. The elevated SOD activity in the roots may be connected to the imbalance of cell oxidation by ER stress. However, no increase in SOD activity was found in the shoots, although CAT activity was increased under tunicamycin-induced ER stress [8]. Therefore, the response procedure should be further studied according to the specific function and localization of these enzymes. Moreover, heterologous overexpression of *Puccinellia tenuiflora APX* can significantly improve salt stress resistance and reduce lipid peroxidation in Arabidopsis [66].

### 4.3. The Redox Systems in the ER

As we discussed in the context of oxidative protein folding above, ER redox homeostasis is essential for protein folding and disulfide bond formation, because they are highly sensitive to the redox status. Therefore, the redox balance is highly maintained by various redox systems such as the GSSG-GSH cycle and PDI response. In particular, ERO-PDI mechanisms, as parts of the UPR, can be prominently induced by ER stress to form the required disulfide, resulting in ERO-mediated H_2_O_2_ production increases [4]. The transcription levels of six Arabidopsis *PDI* genes were found to be activated by ER stress as part of the UPR [67]. However, four of these genes *AtPDI1-2*, *AtPDI4-2*, *AtPDI5-1*, and *AtPDI5-2* were down-regulated in the Arabidopsis *bzip60* mutant, indicating that AtbZIP60, a key transcription factor of the UPR, is essential for the upregulation of *AtPDIs* [67]. After PDI obtains electrons from the polypeptide substrate chain, ERO1 helps PDI to be reoxidized, and H_2_O_2_ is generated during the electron transaction [19]. With the increment in potential substrates upon ER stress, the chance of the formation of mismatched disulfides by free thiols in polypeptides increases, thereby further boosting ERO-PDI-mediated H_2_O_2_ generation. Thus, it could be concluded that ERO activity induced by ER stress might benefit oxidative stress, and it is common in animals, yeasts, and plants [4]. Under ER stress, these generated ROS molecules may be transported throughout the cytoplasm and interfere with other organelles, as well as acting on GSH in the ER to increase the level of GSSG, resulting in the imbalance of GSH-GSSG. It was determined in mammals that PERK contributed to eIF2α phosphorylation and activated the expression of the GSH upstream transcription factors *ATF4* and *NF-E2-related factor 2* (Nrf2), leading to GSH production [19]. Further, *PERK*-deficient cells are more susceptible to H_2_O_2_ and ER stress, indicating that PERK is an essential kinase during ER stress as well as GSH production [19]. However, it does not exist in plants [33]. The Ca^2+^ signal has also been reported to link closely with GSH and ROS. In mammals, the thiol oxidation of the ER-localized Ca^2+^ channel ryanodine receptor (RyR) was activated by ROS or GSSG and inhibited by reductants such as GSH [4]. In yeast, when glutaredoxin (GRX6) was lacking, the ER redox status changed to a more oxidized state, affecting intracellular Ca^2+^ balance [68]. Intriguingly, the Ca^2+^ level in the ER of the *grx6* mutant decreased, while the accumulation of Ca^2+^ in the cytoplasm increased [68]. However, these findings have not been found in plants, so it is worth exploring the connection between GSH-GSSG and ROS in plants’ ER.

### 4.4. ROS as Signaling Molecules between the ER and Other Organelles

Under adverse environments, in addition to the ER, ROS originate from plant mitochondria and chloroplasts; the former mainly play a major role in dark conditions or non-green tissues, and the latter produce ROS for electron transfer in photosynthetic mechanisms to prevent antenna overload and subsequent damage [69]. Numerous studies have shown that mitochondria, chloroplasts, and the ER collaborate in various metabolic processes and exchange signaling molecules under stress conditions, mainly ROS. Yeast Ero1α-derived H_2_O_2_ can diffuse freely in and out of a rough ER, indicating that H_2_O_2_ has penetrability in the ER [70]. Another study in yeast reported that increased ROS caused oxidative damage in mitochondria as well as ROS production from the ER surface [71]. Some studies have also suggested that ER-induced oxidative stress can impact mitochondrial ROS generation, which is most likely mediated by physical interactions between the ER and the mitochondria [4]. On the one hand, ROS production in mitochondria is induced and regulated by ER stress; on the other hand, mitochondrial ROS could induce ER stress-related gene expressions, such as the UPR initiation genes *bZIP28* and *bZIP17* [72]. As with mitochondria, the plant-specific cellular structure of chloroplasts is also tightly associated with the ER. Through a trans-organellar complementation assay, the ER membrane was partially fused with the plastid envelope to form a double-layer membrane, which may make ROS pass through the ER-chloroplast membrane connection domain [73]. It was demonstrated that ROS produced from chloroplasts and mitochondria play special functions in inducing the ER stress response in Arabidopsis. For example, the expression patterns of ER stress-related genes induced by ROS from different chemicals were significantly different, including the rotenone treatment of mitochondria, the methyl viologen (MV) and 3-(3,4-dichlorophenyl)-1,1-dimethylurea (DCMU) treatments of chloroplasts, the 3-amino-triazole (3-AT) treatment of peroxisomes, and direct H_2_O_2_ treatment [72]. In addition to mitochondria and chloroplasts, the ER is closely related to peroxisomes and is involved in their biogenesis. Peroxisomes dynamically expand along the route established by ER tubules in Arabidopsis, and these extensions responded rapidly when exposed to low concentrations of H_2_O_2_ and hydroxyl radicals [74]. Moreover, peroxisomal APX, transported from the ER, contributed to the biogenesis of plant peroxisomes [75]. Overall, this evidence suggests that the ER may transmit and receive redox signals to and from other organelles through the bidirectional permeability of redox compounds and metabolites such as ROS.

### 4.5. The Interplay between ER Stress and ROS during Biotic/Abiotic Stress

Accumulated pieces of evidence have verified the association of ER stress and oxidative stress during various diseases and conditions in mammals, such as cardiovascular disease, bowel disease, liver disease, and diabete, et al. [16,18,76]. In recent years, the interrelation between ER stress and oxidative stress in plants has also gradually attracted more attention. There is much crosstalk between oxidative stress and ER stress, both of which are involved in a range of plant physiological and molecular processes and are induced by common biotic or abiotic stresses. Taking heat stress as an example, plants exposed to high heat would rapidly generate and accumulate ROS in various cellular compartments, resulting in oxidative damage, adverse effects on plant growth, and risks to plant development [77]. As mentioned above, heat stress can cause ER stress in plants. As key transcription factors in response to ER stress, Arabidopsis *bZIP17* and *bZIP28* were highly up-regulated under heat stress [78]. The heating temperature could increase the translocation of these two *bZIPs* to the nucleus and regulate downstream stress response genes [78]. Their single null mutants were more sensitive to 42 °C heat stress [79]. Meanwhile, one previous study also showed that Arabidopsis *bzip28-1* and *bzip28-2* T-DNA insertion mutants did not show significant differences in growth and physiology from those of the wild-type plants when exposed to 40 °C for four days, indicating that other heat response pathways might be triggered to compensate for the lack of *bZIP28* [80]. The study demonstrated that under heat stress, the *bZIP28*-deficient plants exhibited a higher level of APX proteins and promoted H_2_O_2_ accumulation, as well as activating HSPs-dependent pathways with a higher number of *HsfA2* transcripts [80]. These results indicate that plants have flexible response pathways to heat stress, including bZIP28, HSFA2, and ROS-dependent signal transduction. Similarly, salinity can also induce the translocation of bZIP17 and bZIP28 to the nucleus, and there may be similar pathways to dynamically regulate ROS and ER stress. Additionally, Arabidopsis bZIP28 was reported to interact with the CCAAT box binding factor and the nuclear factor-Y (NF-Y) transcription factor, both of which were up-regulated under ER stress and formed a complex to regulate the expression of downstream stress-related genes [81]. Additionally, it was reported in mammals that NF family transcription factors are closely related to ROS, such as NF-κB, which can directly affect ROS levels by increasing the expression of antioxidant proteins [82]. Through a chromatin accessibility assay, an ATAC-seq experiment identified that NF-Y transcription factors could respond to ROS changes in Arabidopsis [83]. One recent study reported the transcription and translation of the key transcription factors bZIP28, bZIP60, and NF-YC2 in pollen tubes by in vitro pollen germination experiments and further showed that these genes responded to heat stress via transcriptome analysis [84,85]. These results further suggested that bZIPs might interact with other key transcription factors, such as the NF family related to ROS signaling, to regulate the ER stress and oxidative stress response under abiotic stress.

In addition to abiotic stress, ER stress can also respond to biological factors. For example, during plant RNA virus infection, the overaccumulation of viral proteins and changes in the lipid membrane structure cause ER stress in plant cells [86]. When *Nicotiana benthamiana* was infected with the *Rice stripe virus*, the NbbZIP17, NbbZIP28A, and NbbZIP28B proteins in the ER stress signaling pathway were activated and expressed [87]. In addition, when *Arabidopsis thaliana* leaves were infected with *Turnip mosaic virus* (TuMV), bZIP60 significantly accumulated after infection compared with the control, indicating that the splicing of *bZIP60* mRNA was activated and downstream ER stress-related genes were up-regulated, such as *BiP3*, *BiP1/2,* and *PDI* [88]. When considering the crosstalk between ER stress and ROS in biotic stress, the key plant hormone salicylic acid (SA) has to be involved. In general, in the case of biotic stress, SA plays an important role in transmitting pathogen signals and activating defense responses [89]. For instance, the endogenous accumulation of SA was induced by TuMV infection in cabbage leaves, and the treatment of the leaves with exogenous SA could induce TuMV resistance, which could play a role in suppressing viral replication [90]. Moreover, SA plays a dual role in the regulation of ROS, which, at a low concentration, benefits ROS production and, at a high level, activates ROS scavenging [91]. These observations logically help to assume there is a possible association among SA, ROS, and ER stress. Furthermore, a negative regulator of SA, constitutive expresser of pathogenesis-related genes 5 (CPR5), was discovered to be a negative regulator of bZIP28 and bZIP60 via protein interactions, and ER stress-related genes dependent on the IRE1-bZIP60 and bZIP28 pathways were induced in the Arabidopsis *cpr5* mutant [92]. Therefore, CPR5 regulates ER stress resistance and plays a role in the SA-mediated homeostasis of plant growth. However, it is still difficult to distinguish their complex correlation among SA, ROS, and ER stress considering those dynamic flexible processes. Therefore, SA, as a key node factor in regulating ER and ROS signal transduction processes, the temporal order, and spatiality of the regulation process, remains to be further explored. Moreover, the real living environment of plants is often more complex, especially ER stress and oxidative stress as secondary stress responses that may be induced by multiple factors at the same time. Thus, the stress responses elicited by the limitation of a single environmental component have been interrelated with other restrictions. The response of plants to multi-factor stresses is more complicated. It has been shown that the abiotic stress factors heat and drought significantly altered the TuMV biotic stress signaling in Arabidopsis, resulting in the inactivation of defense responses and higher plant susceptibility [93]. Therefore, it is worth discussing to distinguish the response of ER stress and oxidative stress to different simultaneous stimuli.

## 5. Conclusions and Perspectives

Although the role of ER stress and its connection with other metabolic processes such as ROS have been well documented in mammals, especially in different diseases, the role of ER stress-related ROS production and signal transduction in plants remains to be further studied. Similar to animal systems, the ER provides a space for oxidative protein folding which produces H_2_O_2_ [16]. When facing different abiotic and biotic stress environments, plants accumulate a large number of unfolded or misfolded proteins which do not have a particular function in the ER lumen, leading to ER stress [4]. Additionally, ER stress can benefit ROS generation and even cause oxidative burden in plants, finally leading to oxidative stress depending on the degree of ER stress. Furthermore, ER stress activates NADPH oxidase-mediated ROS signaling, increases antioxidant defense mechanisms, and alters the GSH redox state. In addition to the ER, accumulating evidence supports that a large amount of ROS can be produced from different plant cellular compartments under stress conditions, including mitochondria and chloroplasts, triggering the UPR [27]. These results suggest that ER stress and ROS in plants have reciprocal crosstalk, which may be seen as a positive feedback mechanism [4]. Additionally, it can be concluded that ER stress, as well as oxidative stress, is secondary stress generated by adverse environments, and these two stresses are directly or indirectly linked with other signaling pathways. However, given the complexity and variability of these processes, some issues remain to be addressed, such as the following: How do plants’ ER and ROS respond when exposed to various stimuli simultaneously? Are there more key node factors in plants to regulate both the ER response and ROS signaling process? How do other second messengers play roles with ROS in ER stress? Besides ROS, do RNS and their derived complexes function under ER stress? In summary, understanding the relationship between ER stress and ROS is an important future challenge, and an in-depth understanding of the role of ROS in ER stress and the UPR will help to design strategies to alleviate ER stress and improve crop survival in agricultural applications.
